# Secondary bladder amyloidosis with familial Mediterranean fever in a living donor kidney transplant recipient: a case report

**DOI:** 10.1186/s13104-016-2273-2

**Published:** 2016-10-19

**Authors:** Sentaro Imamura, Shintaro Narita, Ryuta Nishikomori, Hiroshi Tsuruta, Kazuyuki Numakura, Atsushi Maeno, Mitsuru Saito, Takamitsu Inoue, Norihiko Tsuchiya, Hiroshi Nanjo, Toshio Heike, Shigeru Satoh, Tomonori Habuchi

**Affiliations:** 1 Department of Urology, Akita University School of Medicine, 1-1-1 Hondo, Akita, 010-8543 Japan; 2 Department of Pediatrics, Kyoto University Graduate School of Medicine, Kyoto, Japan; 3 Department of Pathology, Akita University Hospital, Akita, Japan; 4 Center for Kidney Disease and Transplantation, Akita University Hospital, Akita, Japan

**Keywords:** Bladder amyloidosis, Familial Mediterranean fever, Macroscopic hematuria, Renal transplantation

## Abstract

**Background:**

Secondary bladder amyloidosis is an extremely rare disease, resulting from a chronic systematic inflammatory disorder associated with amyloid deposits. Although uncommon in Japan, familial Mediterranean fever (FMF) is a hereditary autoinflammatory disease characterized by recurrent episodes of fever of short duration and serositis and is frequently associated with systemic amyloidosis. Here, we present a case of a Japanese patient complaining of fever and macroscopic hematuria after a living donor renal transplantation. Consequently, he was diagnosed with secondary bladder amyloidosis with FMF.

**Case presentation:**

A 64-year-old Japanese male received a living ABO-incompatible kidney transplant from his wife. The postoperative clinical course was normal, and the patient was discharged 21 days after the transplantation with a serum creatinine level of 0.78 mg/dl. The patient frequently complained of general fatigue and fever of unknown origin. Six months later, the patient presented with continuous general fatigue, macroscopic hematuria, and fever. Cystoscopic examination of the bladder showed an edematous region with bleeding, and a transurethral biopsy revealed amyloid deposits. His wife stated that the patient had a recurrent high fever since the age of 40 years and that his younger brother was suspected to have a familial autoinflammatory syndrome; thus, the patient was also suspected to have a familial autoinflammatory syndrome. Based on his brother’s medical history and the genetic tests, which showed a homozygous mutation (M694V/M694V) for the Mediterranean fever protein, he was diagnosed with FMF. Although colchicine treatment for FMF was planned, the patient had an untimely death due to heart failure. We re-evaluated the pathological findings of the various tissue biopsies obtained during the treatment after the renal transplantation. Immunohistochemistry revealed amyloid deposits in the bladder region, renal allograft, and myocardium and the condition was diagnosed as AA amyloidosis associated with FMF.

**Conclusion:**

We presented a case of systemic amyloidosis with FMF, involving the bladder region, myocardium, and renal allograft, diagnosed after renal transplantation. Bladder amyloidosis should be considered in patients with macroscopic hematuria, particularly in the kidney transplant recipients with idiopathic chronic renal disease. Diagnosis of secondary bladder amyloidosis may result in the early detection of underlying diseases, which may contribute to patient prognosis.

## Background

The amyloidoses contribute a large group of diseases associated with misfolding of extracellular protein [1]. Bladder involvement in amyloidosis is rare; and the involvement is mostly restricted to the bladder, which is called primary bladder amyloidosis [2]. Secondary bladder amyloidosis has been reported in patients with rheumatoid arthritis, Crohn’s disease, ankylosing spondylitis, multiple myeloma, and familial Mediterranean fever (FMF) [2]. FMF is a hereditary autoinflammatory disease characterized by recurrent episodes of fever of short duration and serositis, and it is frequently associated with systemic amyloidosis [3]. According to a nationwide survey, it is an uncommon disease that affects approximately 300 patients in Japan [4].

Here, we report a rare case of secondary bladder amyloidosis as a part of systemic amyloidosis associated with FMF in a Japanese patient who underwent a living donor renal transplantation.

## Case presentation

**Fig. 1:**
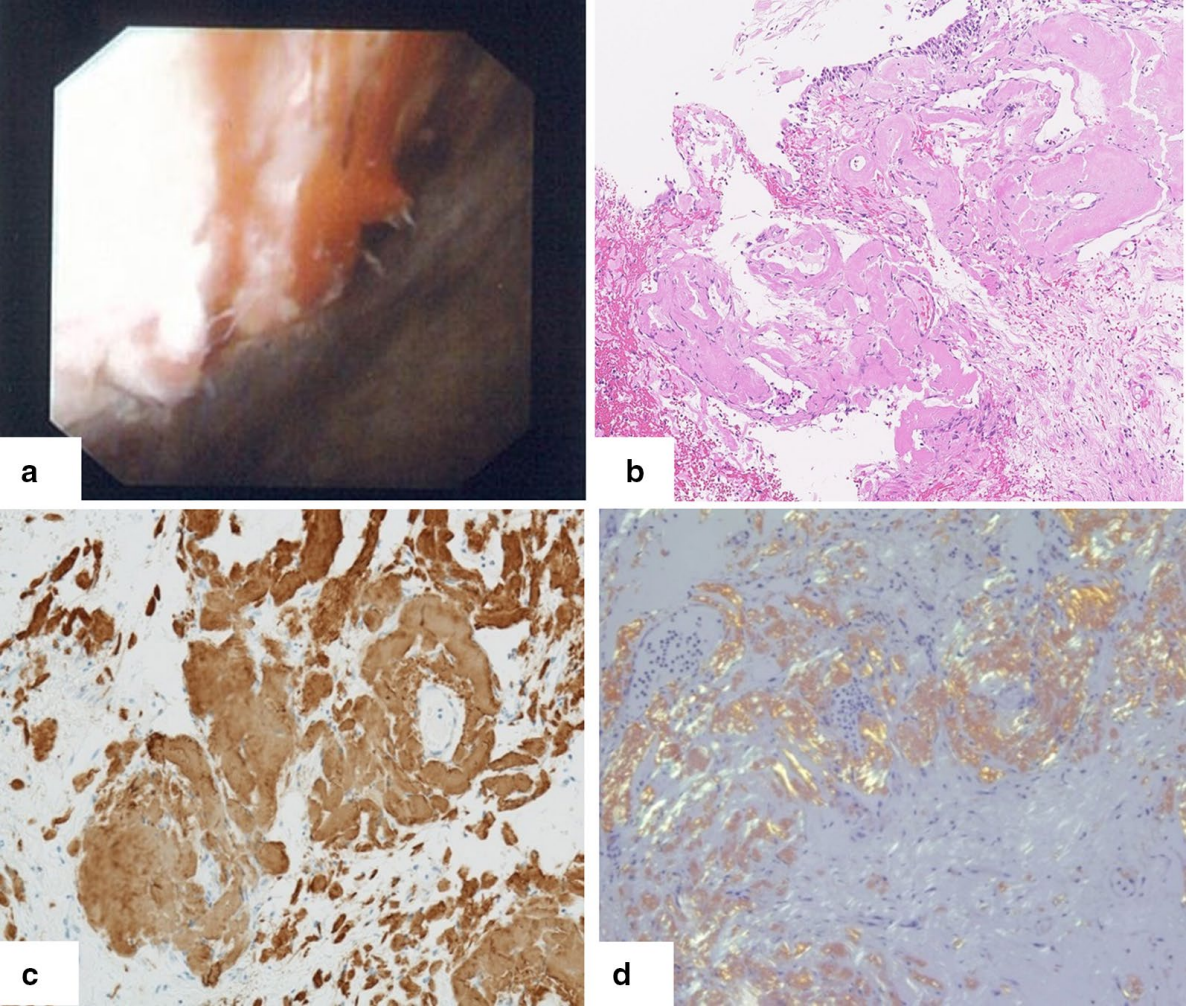
**a** Cystoscopic examination revealed edema and bleeding in the left anterior region of the bladder. **b** Hematoxylin–eosin staining of the amorphous structure showed no malignant cells and diffuse homogenous deposits. **c** Immunostaining for AA amyloidosis was positive in bladder biopsy. **d** *Congo red* staining under polarized light was positive in bladder biopsy

**Fig. 2:**
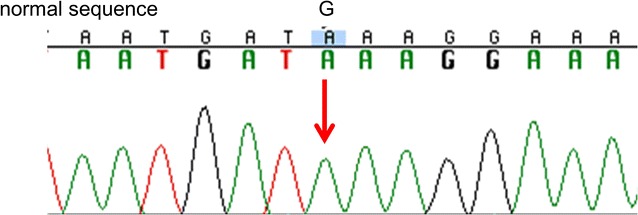
DNA sequence electropherograms demonstrating the M694V mutation of the Mediterranean fever (MEFV) gene

A 64-year-old pure Japanese male with end-stage idiopathic chronic kidney disease, scheduled to receive a living ABO-incompatible renal transplant from his wife, was referred to our renal transplant center in April 2012. His past medical history included angina pectoris and cholecystitis. Physical and laboratory findings were normal, except for the abnormal findings related to end-stage renal disease. The protocol for antibody removal before the transplant consisted of double-filtration plasmapheresis and rituximab. The induction immunosuppressive regimen included a combination of prolonged-release tacrolimus, mycophenolate mofetil, methylprednisolone, and basiliximab. Renal transplantation was performed in December 2012. The postoperative clinical course was normal, and the patient was discharged 21 days after the renal transplantation, with a serum creatinine level of 0.78 mg/dl. Three months after the renal transplantation, the patient was admitted to our hospital with fever and general fatigue. Clinical examination, including chest and abdominal computed tomography, blood and urine culture, and cardiac ultrasound, revealed no abnormal findings, and the symptoms improved after antibiotic treatment. Three months later, the patient complained of fever and macroscopic hematuria. Cystoscopic examination revealed edema and bleeding in the left anterior region of the bladder (Fig. 1a). Because cold biopsy of the abnormal mucosa showed no malignant cells on hematoxylin–eosin staining (Fig. 1b), at that time, the bladder abnormality was suspected to be caused by viral hemorrhagic cystitis. The macroscopic hematuria stopped 3 days after dose reduction of the immunosuppressive drugs. Subsequently, the patient presented with intermittent fever, which resolved spontaneously within a few days. In May 2014, he presented with fever, general fatigue, and urinary retention and was re-admitted to our hospital. Ten days after admission, he required an artificial respirator and was transferred to an intensive care unit in circulatory shock. Because the precise cause for the aggravation of his general condition, frequent episodes of fever, and continuation of general fatigue was not clarified, his wife offered more details about the patient’s medical history. She stated that he had recurrent high fever since the age of 40 years and that his younger brother was suspected to have a familial autoinflammatory syndrome. After written informed consent, the patient’s serum sample was sent to the Department of Pediatrics at the Kyoto University Graduate School of Medicine for screening mutations and polymorphisms associated with hereditary autoinflammatory diseases. The ethics committee of the Akita University School of Medicine approved the use of human samples in the present study. During waiting the results of the genetic test, the patient continued rehabilitation at a related hospital. However, 2 months after discharge from our hospital, he presented with fever and heart failure and was re-admitted. At that time, he was found to be homozygous for the Mediterranean fever (MEFV) mutation (M694V/M694V) (Fig. 2). Based on the presence of the MEFV mutation and the patient’s history, he was diagnosed with FMF. Treatment with colchicine was planned, but the patient died from heart failure in October 2014. We re-evaluated the pathological findings of the various tissue biopsies obtained during his treatment after the renal transplantation because FMF is known to be associated with systemic amyloidosis. Immunohistochemistry revealed amyloid deposits in the abnormal bladder region (Fig. 1c, d), transplanted kidney, and myocardium, which was diagnosed as AA amyloidosis associated with FMF.

## Discussion

Hematuria has a prevalence of 12 % in the post renal transplant patients with the major causes such as urinary tract infection, genitourinary malignancies, graft rejection, recurrences of primary disease and calculus [5]. Although bladder amyloidosis mainly presented intermittent painless macroscopic hematuria [6], we had not considered bladder amyloidosis at the time of the presence of macroscopic hematuria because of its rare cause of macroscopic hematuria in post renal transplant patients.

Amyloidosis is mainly classified into primary (AL) or secondary (AA), according to the biochemical nature of the protein forming the fibril [2]. The secondary bladder amyloidosis incidence is lower than primary localized bladder amyloidosis [7]. Secondary bladder amyloidosis is often associated with a chronic inflammatory disease; only 30 cases have been previously reported [2]. One patient presented with secondary bladder amyloidosis with FMF in a large study of systemic amyloidosis and FMF [8]. To our knowledge, this is the second case of secondary amyloidosis associated with FMF and the first case of secondary bladder amyloidosis in an FMF patient treated with renal transplantation. Although a rare cause of macroscopic hematuria in renal transplant patients, the presence of secondary bladder amyloidosis associated with a systemic disease should be considered, particularly in renal transplant recipients with idiopathic chronic kidney disease.

FMF is a disease associated with a mutation in the MEFV gene, which is located on chromosome 16 and encodes a 781-amino-acid protein named pyrin [9]. This protein may play a pivotal role in inflammation and apoptosis regulation [3]. In a study on renal transplantation in FMF patients [10], during a mean follow-up of 35 months, all allografts were functioning well and the patients’ mean serum creatinine level was 1.4 mg/dl. Because the outcome after renal transplantation is mainly influenced by cardiac disease severity [11], the Canadian Society of Transplantation recommends renal transplantation for patients with amyloidosis if there is no evidence of cardiac involvement [11]. Before renal transplantation, we could not detect any cardiac problem on the electrocardiogram, chest radiograph, or cardiac ultrasound, except for a mild tricuspid valve insufficiency. We should be careful about abnormal findings related to systemic amyloidosis in patients with renal transplantation during routine screenings and post-transplantation follow up. Furthermore, if a kidney biopsy had been performed to elucidate the cause of the idiopathic chronic kidney disease, amyloidosis may have been diagnosed at an earlier stage. It is also important to identify the cause of idiopathic chronic kidney disease before renal transplantation, particularly in patients with symptoms of unknown origin and familial history.

The main treatment strategy for secondary amyloidosis is control of underlying disease. As an antigout and antimitotic agent that decreases leukocyte motility and phagocytosis in inflammatory responses, continuous colchicine use is recommended to prevent frequent attacks and amyloidosis in FMF [12]. Colchicine administration may be helpful in preventing further complications and improving the prognosis in our patient.

## Conclusion

We presented a case of FMF with systemic amyloidosis involving the bladder, myocardium, and renal allograft diagnosed after the renal transplantation. The patient died of heart failure because of cardiac amyloidosis without receiving any treatment for FMF. Although bladder amyloidosis associated with FMF is rare, it should be considered in patients with gross hematuria, particularly renal transplant recipients with idiopathic end-stage renal disease.

## Abbreviations

FMF: familial Mediterranean fever
MEFV: homozygous for the Mediterranean fever
